# Index of contractile asymmetry improves patient selection for CRT: a proof-of-concept study

**DOI:** 10.1186/s12947-019-0170-2

**Published:** 2019-10-10

**Authors:** Tomas Zaremba, Bhupendar Tayal, Sam Riahi, Anna Margrethe Thøgersen, Niels Eske Bruun, Kasper Janus Grønn Emerek, Joseph Kisslo, Thomas Fritz Hansen, Niels Risum, Peter Søgaard

**Affiliations:** 1 Department of Cardiology, Aalborg University Hospital, Hobrovej 18-22, 9100 Aalborg, Denmark; 2 Department of Clinical Medicine, Aalborg University, Sdr. Skovvej 15, 9000 Aalborg, Denmark; 3 Institute of Clinical Medicine, Copenhagen University, Blegdamsvej 3B, 2200 Copenhagen N, Denmark; 4 Zealand University Hospital, Roskilde, Sygehusvej 10, 4000 Roskilde, Denmark; 5 Division of Cardiovascular Disease, Duke University Medical Center, 2301 Erwin Rd, Durham, North Carolina 27710 USA; 6 Department of Cardiology, Herlev-Gentofte University Hospital, Kildegårdsvej 28, 2900 Hellerup, Denmark; 7 Department of Cardiology, Copenhagen University Hospital Rigshospitalet, Blegdamsvej 9, 2100 Copenhagen, Denmark

**Keywords:** Strain rate, Heart failure, Cardiac resynchronization therapy, Contractile asymmetry

## Abstract

**Background:**

Nearly one-third of heart failure (HF) patients do not respond to cardiac resynchronization therapy (CRT) despite having left bundle branch block (LBBB). The aim of the study was to investigate a novel method of quantifying left ventricular (LV) contractile asymmetry in HF.

**Methods:**

Patients with HF and LBBB undergoing CRT (*n* = 89, 37.1% females, 68 ± 9 years, ischemic etiology in 61%, LV ejection fraction 27.1 ± 7.1%) were analyzed. LV longitudinal systolic strain rate values were extracted from curved anatomical M-mode plots of standard long-axis 2D-echocardiography images and cubic spline interpolation was used to generate a 3D-phantom. Index of contractile asymmetry (ICA) was calculated based on standard deviation of differences in strain rate of opposing walls. Average ICA was individually assessed pairwise in 12 opposing 30-degree LV sectors. Reduction in LV end-systolic volume (ESV) ≥15% after 6 months was considered as positive response to CRT.

**Results:**

CRT response was found in 66 (74.2%) patients. Responders with both ischemic and non-ischemic cardiomyopathy had a higher and more extensive contractile asymmetry at baseline and achieved a greater ICA reduction after CRT than non-responders. Higher baseline ICA predicted higher degree and wider extent of ICA improvement. Also, both ICA at baseline and reduction of ICA correlated with the degree of ESV reduction after CRT.

**Conclusions:**

Quantification of asymmetrical LV activation in 3D by ICA provides valuable insights into LV contraction in case of LBBB and is a promising tool for improved patient selection for CRT.

## Background

Cardiac resynchronization therapy (CRT) is an established treatment in symptomatic systolic heart failure (HF) patients and the strongest body of evidence is seen in patients with left bundle branch block (LBBB). However, despite pre-selection by ECG morphology, the number of non-responders is disappointingly high.

Assessment of mechanical dyssynchrony by echocardiography provides incremental prognostic information both pre- and post-implantation [1–8], in particular when assessing dyssynchrony patterns compatible with an underlying conduction disorder [3–7]. However, there are still remaining issues to be resolved, such as precise location of dyssynchrony for optimal lead positioning during implantation as well as impact of resynchronization in the left ventricular (LV) lead target area to restore a near normal contraction pattern.

Currently used tissue Doppler imaging (TDI) and speckle tracking echocardiography (STE) techniques deduct the presence of LV mechanical dyssynchrony from a stringently limited number of curves. Despite increasingly high spatial resolution, the deformation data in 2D echocardiography in such case are reduced to two or at most six curves per view, while in 3D, LV dyssynchrony is usually judged from 16 deformation curves based on a 16-segment LV model [9].

The aim of this proof-of-concept study was to define the impact of the three-dimensional localization and magnitude of STE-based LV contractile asymmetry of entire opposing walls on improvement in LV function after CRT in patients with HF and LBBB.

## Methods

The study was conducted as an analysis of prospectively acquired data in patients with HF having LBBB who underwent implantation of CRT device at two centers: Aalborg University Hospital and Gentofte University Hospital. Only the patients fulfilling ECG criteria for typical LBBB and QRS duration ≥ 120 ms were included [10]. Follow-up period was at least 6 months after the CRT implantation. Positive response to CRT was defined as a reduction of LV end-systolic volume (ESV) by ≥ 15% compared to baseline echocardiography [11–13]. In total, 89 patients were included (Aalborg *n* = 49, Gentofte *n* = 40). In addition, echocardiograms from 10 healthy volunteers aged ≥ 18 years with no previous cardiac history were used as controls.

### Clinical characteristics

Medical records of the patients were reviewed manually. Following pre-implantation data were collected: New York Heart Association class, standard heart failure and lipid-lowering medical therapy, renal function, and QRS duration on the 12-lead ECG. Chronic kidney disease was defined as estimated glomerular filtration rate below 60 ml/min/1.73 m^2^ body surface area. Ischemic etiology of cardiomyopathy was defined as having a previous diagnosis of an acute coronary event, having undergone a revascularization procedure, or having a significant coronary artery stenosis (> 70%).

### Image analysis

#### Strain rate

The longitudinal strain rate analysis was performed on ECG-gated 2D-echocardiography images using EchoPAC (r) software version 201 (GE Healthcare, Milwaukee, WI). Two-chamber, three-chamber, and four-chamber 2D images acquired at a mean frame rate of 67.4 ± 11.1 s^− 1^ were analyzed. The duration of systole was defined as the period from the onset of QRS complex to the aortic valve closure in trans-aortic continuous wave Doppler trace. Afterwards, a regular STE-based strain rate analysis was performed.

**Fig. 1:**
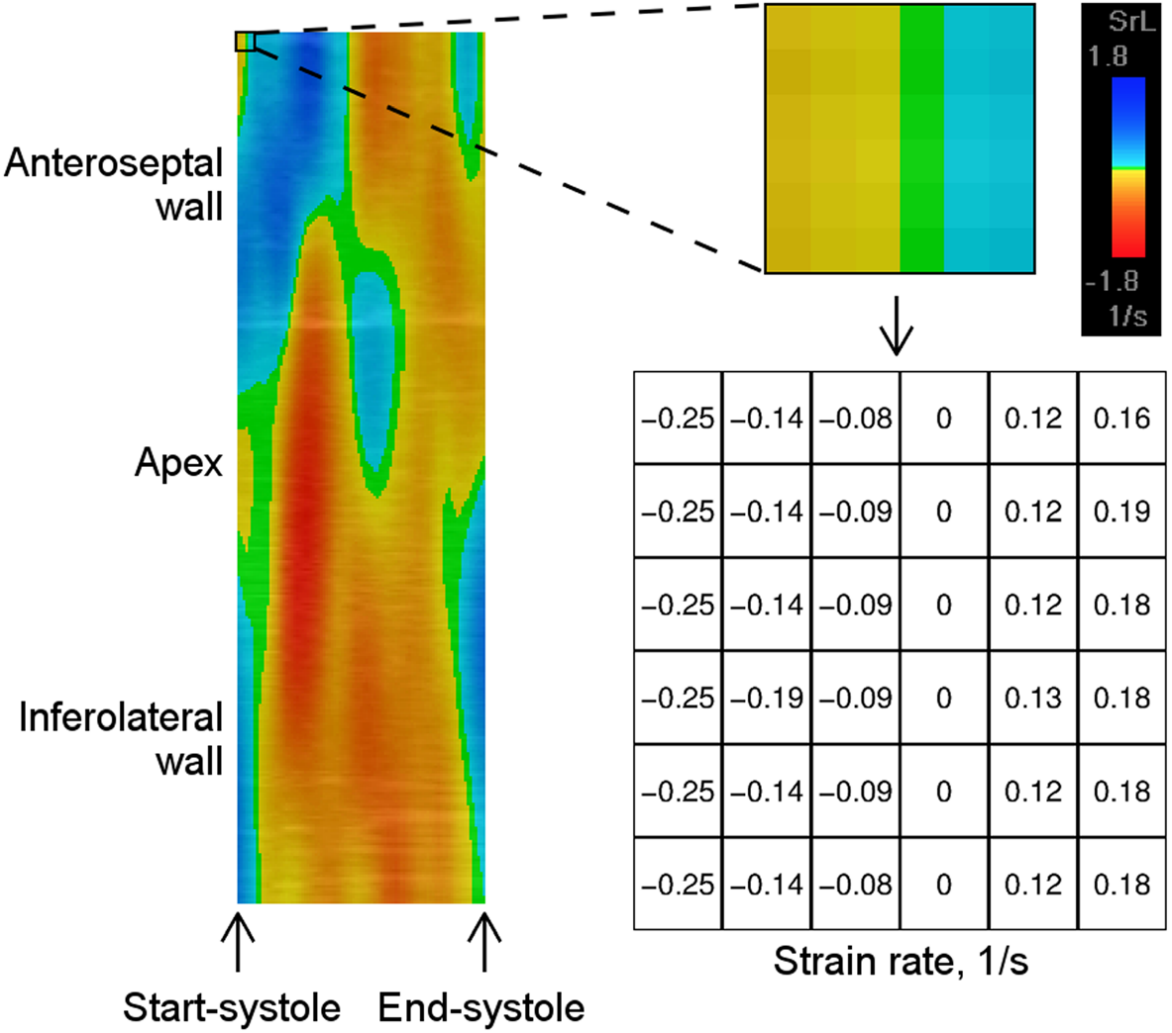
Extraction of strain rate values data from the curved anatomical M-mode plot

The first step of the present fully automated approach was to extract the strain rate data during systole from the curved anatomical M-mode (CAMM) plots (Fig. 1). Standard echocardiography analysis packages such as EchoPAC (r) usually export quantitative data of six segments per view. A strain rate CAMM plot is a rectangular image depicting a strain rate value in each pixel. CAMM plots, which are used for visual representation of strain rate propagation typically contain 330 data-lines per view (the vertical dimension of the CAMM plot). Each of the pixels of the systolic part of a CAMM plot was converted to a strain rate value. For this decoding, the color scale provided in the upper part of EchoPAC (r) display was used. The automated algorithm of the conversion process is outlined in Additional file 1.

The resulting individual strain rate values from each pixel were then arranged in the same order as the corresponding pixels in the CAMM plot. This gives a table of strain rate values, which has the same number of rows and columns as the vertical and the horizontal dimensions of the systolic CAMM plot. As in the original CAMM plot, the upper and the lower halves of the resulting table represent the strain rate from the two opposing walls of the LV, while the LV apex is “located” between the two halves. The start-systole is on the left-hand side, and the end-systole is on the right-hand side of the table.

Next, a 3D model of strain rate throughout the systole was constructed (Additional file 1). Data from the above tables of the three standard apical projections were used. Looking at the LV from the apex and setting the anterior portion of the LV to 0 degrees (12 o’clock), two-chamber, four-chamber, and three-chamber views cover the radii of 0–180, 60–240, and 120–300 degrees, respectively. Thus, the zero-degree mark is set at the anterior LV wall in the two-chamber view, and the scale goes clockwise. Using spline interpolation, a total of 180 apical views were generated, each represented by an individual table containing strain rate values as in standard apical views.

**Fig. 2:**
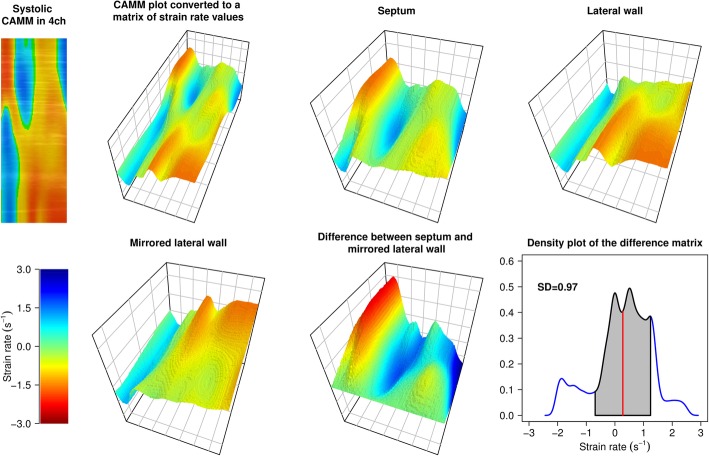
Flowchart of the calculation of index of contractile asymmetry (ICA). Left upper panel: the original curved anatomical M-mode (CAMM) plot of pre-implantation systolic strain rate in an apical four-chamber view in a responder to CRT. The strain rate values contained in the CAMM plot are converted to a table (matrix). ICA is then calculated as standard deviation of the differences in strain rate in two opposing left ventricular walls (here, septum and lateral wall). Left lower panel: scale used in the graphical representation of the strain rate tables (matrices). Right lower panel: density plot of the differences in strain rate between the two walls. In this case, ICA in four-chamber view is 0.97 s^− 1^. 4ch, four-chamber view; CAMM, curved anatomical M-mode; SD, standard deviation

In matrix algebra, a subtraction of two tables (matrices) is performed by element wise subtraction of the corresponding elements, which results in a new matrix of the same dimensions. Hence, the two geometrically opposing upper and lower halves of the strain rate table were subtracted from each other. Here, all symmetrical table entries of the opposing LV walls were subtracted one by one. This provided a new table containing the instantaneous differences in systolic strain rate in the two opposing walls based on the table from each particular view (Fig. 2).

In case of an almost symmetric LV contraction, such resulting table would contain uniform values relatively close to zero. On the other hand, an asymmetric contraction would result in greater strain rate differences of the two opposing walls. Hence, in case of LBBB, an early contraction in one wall (negative strain rate value) and a simultaneous stretch in opposite wall (positive strain rate value) is expected to lead to high absolute values when these symmetrical numbers are subtracted from each other. Subsequently, when the latter wall is activated and the former wall is stretched, high difference values with the opposite sign would be generated. To quantify these asymmetrical counteractions during systole, a standard deviation (SD) of such strain rate differences in the CAMM-plot-derived difference table was calculated both in standard apical views and in each of the remaining 180 views. This value was called index of contractile asymmetry (ICA).

**Fig. 3:**
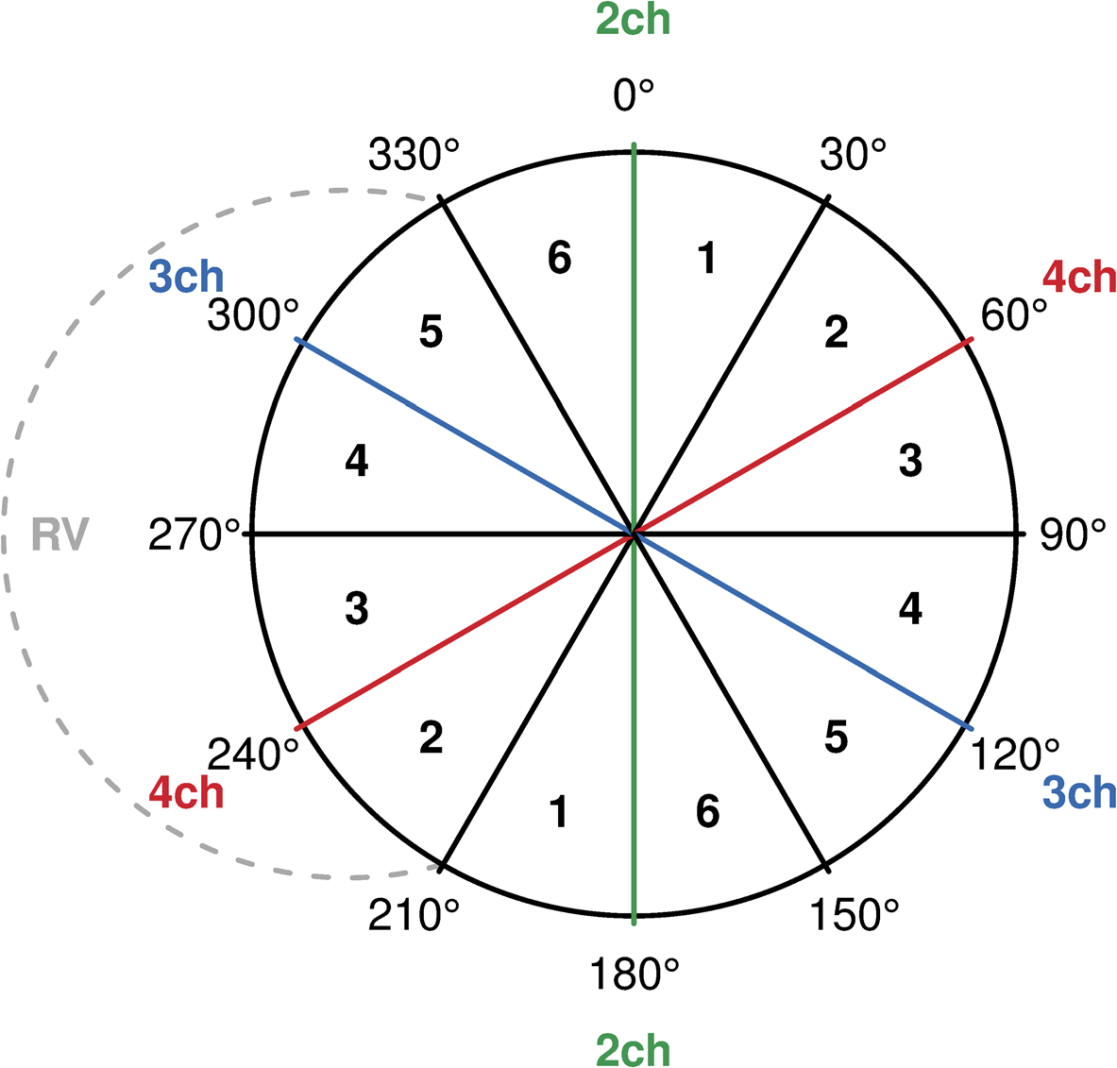
Division of left ventricle into 12 sectors. The circle represents left ventricle (LV) from the bull’s-eye view. The dashed line depicts right ventricle. Zero degrees correspond to the LV anterior wall in the two-chamber view. RV, right ventricle; 2ch, two-chamber view; 3ch, three-chamber view; 4ch, four-chamber view

To assess regional differences in the LV contractile asymmetry, the LV was divided into six sectors, each 30 degrees wide (Fig. 3). Sector 1 included views covering the LV from 1 to 30 degrees in the clock-wise direction and simultaneously crossing the LV in 181 to 210 degrees. Sector 2 covered 31 to 60 degrees (and 211 to 240 degrees), sector 3 covered 61 to 90 degrees with the corresponding opposing angles, etc. An average ICA in each LV sector was calculated, providing six values per LV in each case.

An equivalent strain rate analysis was performed on the post-CRT echocardiograms. Also, the ICA quantification was repeated in a blinded fashion in a random sample of 10 pre-implantation echocardiograms to determine the degree of intra-observer agreement. The repeated analyses were performed in all six LV sector pairs, thus rendering a sample of 60 repeated observations. Intraclass correlation coefficient, coefficient of variation, and bias were calculated.

#### 2D echocardiography and strain

LVEF and LVESV were calculated using the biplane Simpson’s method by a blinded, experienced observer using EchoPAC (r) software. The analyses were performed on the pre-implantation 2D echocardiography images and on the follow-up echocardiography images obtained at least 6 months after the implantation.

Longitudinal strain analysis based on standard apical views was performed on baseline echocardiograms. Global longitudinal strain (GLS) was calculated as a mean of peak strain values in 16 segments. Mechanical dispersion was calculated as an SD of time to peak of longitudinal strain in 16 segments [14]. Mechanical longitudinal strain dyssynchrony was defined as maximum time to peak delay in basal and midventricular segments in the apical four-chamber view using a cut-off of ≥ 130 ms [6, 15]. In addition, pre-implantation echocardiograms were stratified based on presence or absence of classical LBBB pattern [16]. Patients having more than 3 LV segments with inadequate tracking despite manual adjustment were excluded from all STE analyses.

### Statistical analysis

Continuous variables were reported as mean along with their SD. Categorical values were reported as absolute numbers and percentages. Continuous variables were compared either by two-sample Student t-test or by Mann Whitney U test. Fisher exact test was used to compare categorical variables. Linear regression and Pearson’s r were applied to evaluate association between two linear variables. Logarithmic transformation of data was used in case it improved the fit of the model. Receiver operating characteristics (ROC) analysis was used to identify the cutoff and predictive values of ICA. Univariable logistic regression was used to evaluate predictor values selected from clinical perspective in advance. Afterwards, multivariable logistic regression models were built. Two-sided tests were used, and *p* < 0.05 was considered statistically significant. All analyses were performed on R (r) version 3.3.3.

## Results

Demographic and baseline characteristics stratified by CRT response are provided in Table 1. Mean age was 68 ± 9 years, and 33 (37.1%) patients were female. Mean pre-implantation LVEF was 27.1 ± 7.1%, and mean QRS duration was 163 ± 20 ms. According to the defined criteria of LVESV reduction by ≥ 15% after 6 months, 66 (74.2%) patients out of 89 responded to CRT. Responders were near-significantly younger and had a lower prevalence of chronic kidney disease compared to non-responders. QRS duration did not differ between responders and non-responders to CRT (*p* = 0.83).

**Table 1:** Baseline characteristics

	All subjects (*n* = 89)	Responders (*n* = 66)	Non-responders (*n* = 23)	*p*-value
Age, yrs	68 ± 9	67 ± 10	71 ± 8	0.05
Female, n (%)	33 (37.1)	27 (40.9)	6 (26.1)	0.32
Ischemic etiology, n (%)	54 (61)	38 (57.6)	16 (69.6)	0.34
NYHA-class				1
I, n (%)	1 (1.1)	1 (1.5)	0 (0)	
II, n (%)	30 (33.7)	23 (34.8)	7 (30.4)	
III, n (%)	58 (65.2)	43 (65.2)	15 (65.2)	
ACEI/ARB, n (%)	87 (97.8)	64 (97)	23 (100)	0.99
Beta-blockers, n (%)	85 (95.5)	63 (95.5)	22 (95.7)	0.99
Loop diuretics, n (%)	58 (65.2)	40 (60.6)	18 (78.3)	0.2
Aldosterone antagonists, n (%)	49 (55.1)	35 (53)	14 (60.9)	0.63
Statins, n (%)	63 (70.8)	45 (68.2)	18 (78.3)	0.43
eGFR < 60 ml/min/1.73 m^2^, n (%)	38 (42.7)	24 (36.4)	14 (60.9)	0.05
LVESV, ml	132.4 ± 52.9	131.9 ± 56.6	133.9 ± 41.3	0.86
LVEF, %	27.1 ± 7.1	27.3 ± 6.9	26.6 ± 7.7	0.7
GLS, %	−9.5 ± 3.4	−9.7 ± 3.7	−8.7 ± 2.5	0.12
Mechanical dispersion, ms	97 ± 32	97 ± 32	96 ± 34	0.95
QRS duration, ms	163 ± 20	163 ± 18	162 ± 26	0.83

*ACEI* Angiotensin-converting enzyme inhibitors, *ARB* Angiotensin II receptor blockers, *EF* Ejection fraction, *eGFR* estimated glomerular filtration rate, *ESV* End-systolic volume, *GLS* Global longitudinal strain, *LV* left ventricle, *NYHA* New York Heart Association; *, *p* < 0.05

### Pre-implantation ICA

**Fig. 4:**
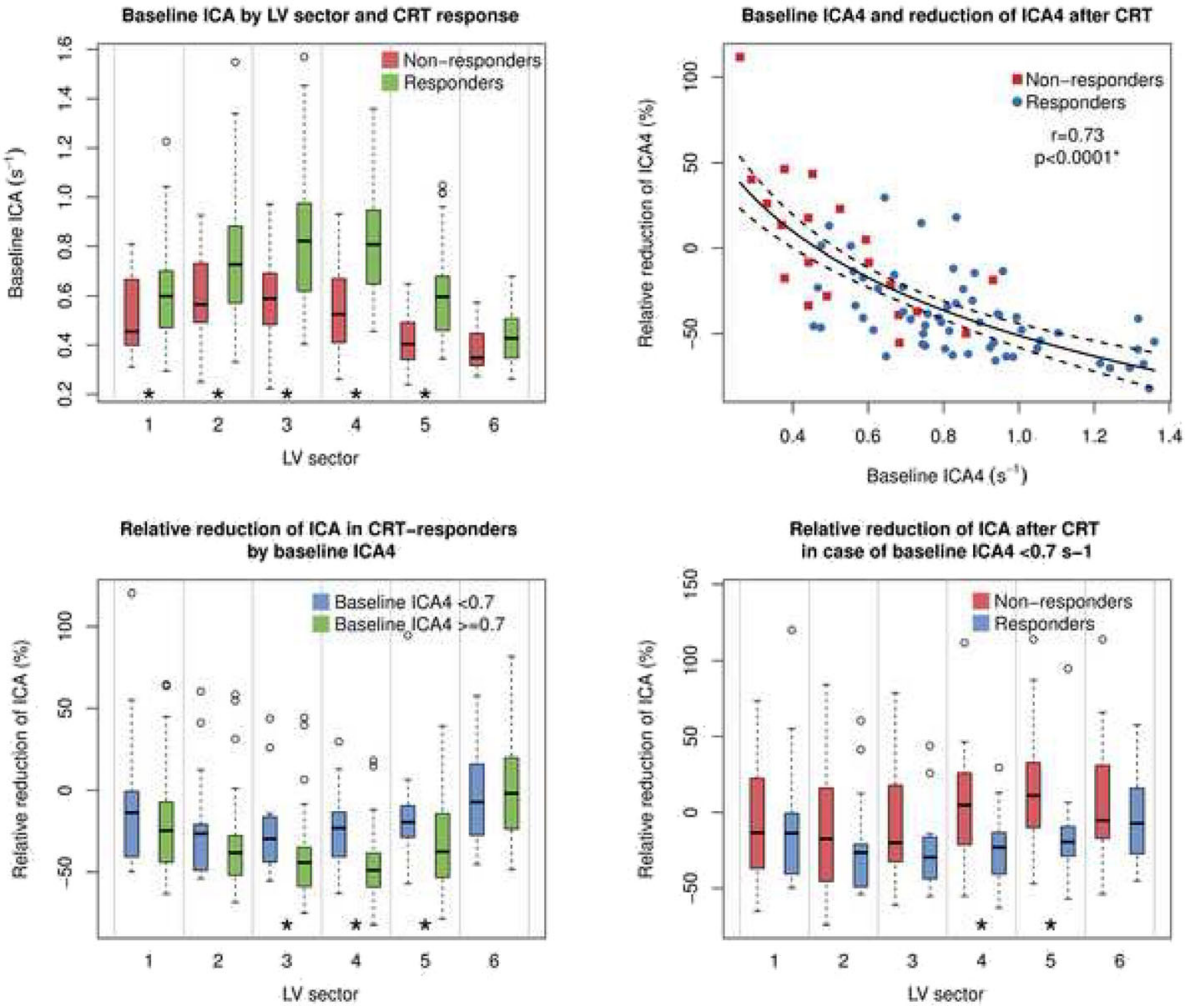
CRT response by ICA in each LV sector. Left upper panel: Baseline ICA in CRT responders vs. non-responders. Right upper panel: Reduction of ICA4 as a function of baseline ICA4. Left lower panel: Reduction of ICA after CRT stratified by the severity of the baseline ICA4. Right lower panel: Reduction of ICA after CRT in the subgroup of patients with a low baseline contractile asymmetry (ICA4 < 0.7 s^− 1^). ICA, index of contractile asymmetry; ICA4, index of contractile asymmetry in LV sector 4; CRT, cardiac resynchronization therapy; LV, left ventricle; r, Pearson’s r; *, *p* < 0.05

**Fig. 5:**
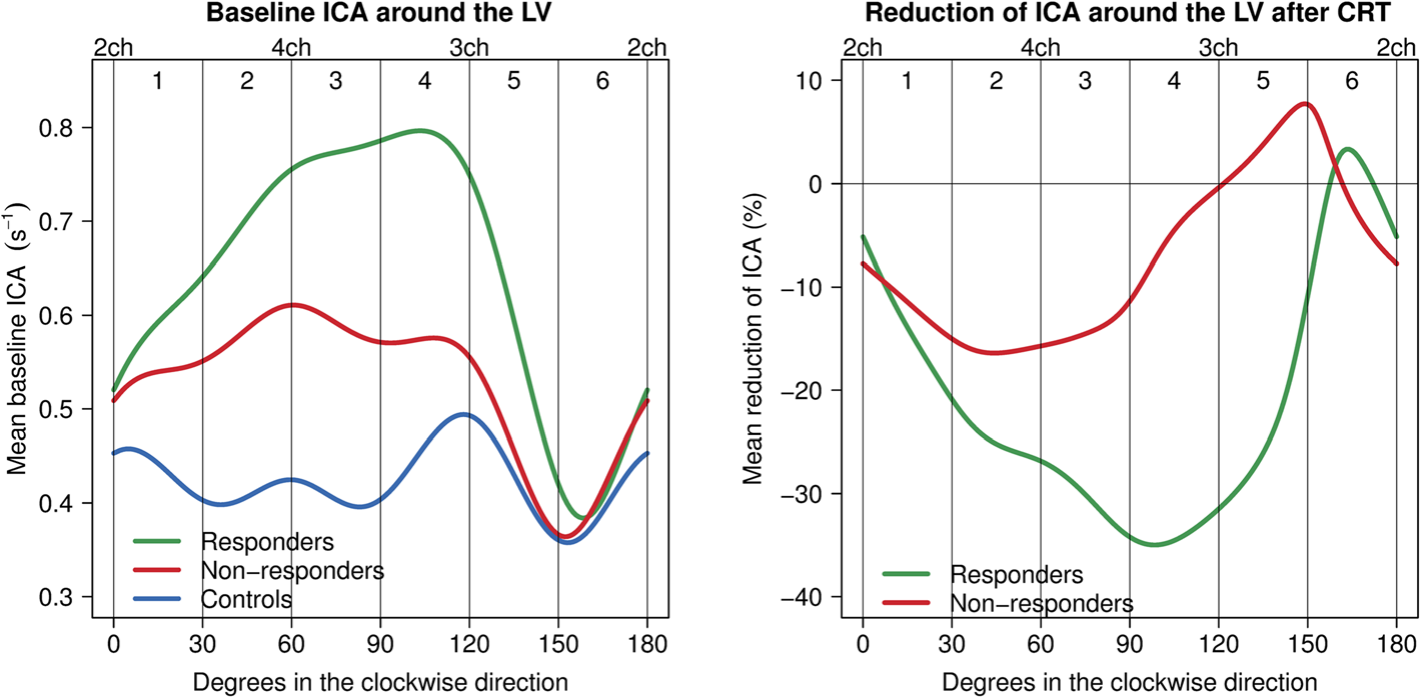
Baseline ICA and reduction of ICA around the left ventricle after CRT. Left panel: Mean baseline ICA in CRT responders, non-responders, and controls in each degree around the LV. Digits 1 to 6 represent the LV sectors. Right panel: Mean relative reduction of ICA after CRT. Abbreviations as in Figs. 3 and 4

An example of traditionally exported strain rate curves from EchoPAC (r) and CAMM plot-generated curves at the identical rows in the CAMM plot are shown in Additional file 2. Responders exhibited a significantly higher ICA on the pre-implantation echocardiograms than non-responders in LV sectors 1 to 5, i.e. in five sectors out of six (Fig. 4 left upper panel). The greatest difference in baseline ICA was seen in LV sector 4 (ICA4), i.e. the average ICA in sector 4 covering degrees 91 to 120 and the opposing 271 to 300: 0.83 ± 0.24 s^− 1^ vs. 0.55 ± 0.18 s^− 1^, *p* < 0.0001. Mean ICA at each angle from 0 to 180 degrees in responders, non-responders, and controls is graphically depicted in Fig. 5. ICA4 was chosen for further analysis. ICA4 in controls (0.46 ± 0.09 s^− 1^) was lower than ICA4 in responders (*p* < 0.0001) but not compared to non-responders (*p* = 0.08) (Table 2).

**Table 2:** Index of contractile asymmetry in standard apical views and six left ventricular sectors at baseline

	All subjects (*n* = 89)	Responders (*n* = 66)	Non-responders (*n* = 23)	*p*-value †	Controls (*n* = 10)	*p*-value ‡
Two-chamber view, s^− 1^	0.52 ± 0.14	0.53 ± 0.15	0.48 ± 0.14	0.14	0.45 ± 0.13	0.6
Three-chamber view, s^−1^	0.71 ± 0.25	0.78 ± 0.23	0.51 ± 0.15	< 0.0001*	0.49 ± 0.12	0.66
Four-chamber view, s^−1^	0.75 ± 0.25	0.79 ± 0.25	0.62 ± 0.18	< 0.001*	0.42 ± 0.11	< 0.001*
ICA1, s^−1^	0.58 ± 0.18	0.61 ± 0.19	0.52 ± 0.16	0.03*	0.44 ± 0.12	0.12
ICA2, s^−1^	0.7 ± 0.23	0.74 ± 0.24	0.59 ± 0.18	0.002*	0.41 ± 0.11	0.002*
ICA3, s^−1^	0.76 ± 0.25	0.81 ± 0.24	0.6 ± 0.19	< 0.0001*	0.41 ± 0.08	< 0.001*
ICA4, s^−1^	0.75 ± 0.26	0.83 ± 0.24	0.55 ± 0.18	< 0.0001*	0.46 ± 0.09	0.08
ICA5, s^−1^	0.56 ± 0.18	0.61 ± 0.18	0.41 ± 0.1	< 0.0001*	0.43 ± 0.13	0.76
ICA6, s^−1^	0.42 ± 0.1	0.44 ± 0.11	0.39 ± 0.1	0.07	0.4 ± 0.13	0.89

*ICA* Index of contractile asymmetry; *, *p* < 0.05; †, responders vs. non-responders; ‡, non-responders vs. controls

**Fig. 6:**
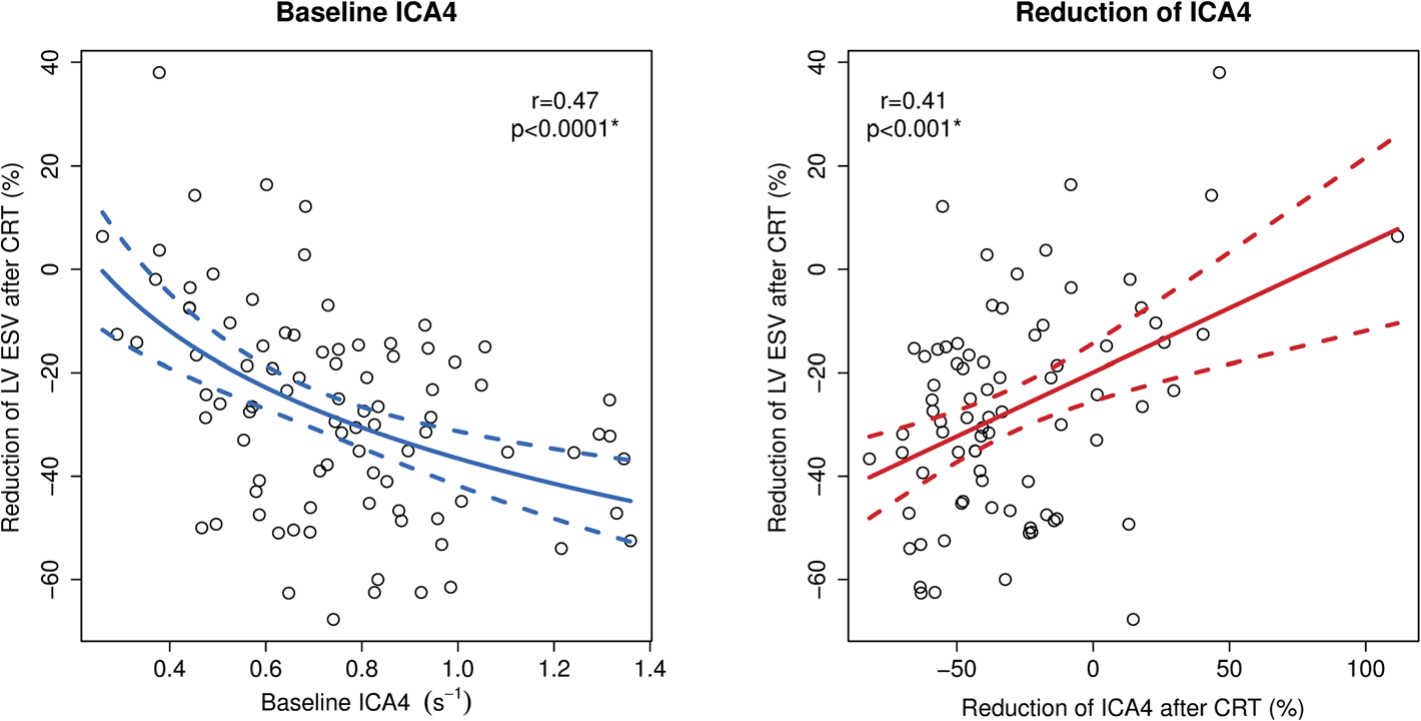
Linear regression of ICA4 and relative reduction of LVESV after CRT. Left panel: Reduction of LVESV after CRT as a function of baseline ICA4. Right panel: Reduction of LVESV after CRT as a function of reduction of ICA4. ESV, end-systolic volume. Other abbreviations as in Fig. 4


ROC analysis of ICA4 for prediction of CRT response yielded an area under the curve (AUC) of 0.83 [95% confidence interval (CI) 0.73–0.93]. A threshold of ICA4 at 0.7 s^− 1^ had sensitivity 71%, specificity 83%, positive predictive value (PPV) 92%, negative predictive value (NPV) 50%, and accuracy 74%. In addition, the reconstruction of 180 radii of strain rate throughout the systole enabled a visual representation of the LV activation from a bull’s-eye view perspective (Additional file 3). A linear relationship between the degree of LVESV reduction after CRT and baseline ICA4 was observed as well (*r* = 0.47, *p* < 0.0001) (Fig. 6 left panel).

**Additional file 3.** Polar plots of dynamic representation of baseline left ventricular strain rate during the systole in a responder and a non-responder to CRT presented as a running loop.


Lowering the cut-off of ICA4 to 0.6 s^− 1^ provided sensitivity 80%, specificity 61%, PPV 85%, NPV 52%, and accuracy 75%. Increasing the cut-off to 0.8 s^− 1^ yielded sensitivity 52%, specificity 91%, PPV 94%, NPV 40%, and accuracy 62%. In the subgroup of patients with ischemic cardiomyopathy, baseline ICA4 had an AUC of 0.82 (95% CI 0.7–0.94), and in non-ischemic cardiomyopathy AUC was 0.83 (95% CI 0.64–1).

### Reduction of ICA after CRT

Post-CRT echocardiograms at 6 months were available for the STE analysis in 79 (88.8%) patients (59 responders, 20 non-responders). Responders had a higher degree of relative reduction of ICA in LV sectors 3 to 5 covering degrees 61 to 150 (2 o’clock to 5 o’clock). In responders, ICA4 decreased by −39 ± 24% vs. 1 ± 41% in non-responders (*p* < 0.0001) (Table 3). There was a linear correlation between the relative reduction of LVESV and the relative reduction of ICA4 (*r* = 0.41, *p* < 0.001) (Fig. 6 right panel).

**Table 3:** Relative reduction of ICA after CRT

	All subjects (*n* = 79)	Responders (*n* = 59)	Non-responders (*n* = 20)	*p*-value
Two-chamber view, %	−4 ± 38	−4 ± 39	−2 ± 39	0.61
Three-chamber view, %	−24 ± 38	−35 ± 29	8 ± 43	< 0.0001*
Four-chamber view, %	−28 ± 34	−33 ± 30	−14 ± 43	0.04*
ICA1, %	−14 ± 35	−16 ± 35	−8 ± 37	0.39
ICA2, %	−26 ± 33	−30 ± 30	−14 ± 41	0.06
ICA3, %	−31 ± 31	−37 ± 26	−13 ± 39	0.005*
ICA4, %	−29 ± 34	−39 ± 24	1 ± 41	< 0.0001*
ICA5, %	−18 ± 37	−28 ± 29	12 ± 42	< 0.0001*
ICA6, %	1 ± 33	−1 ± 31	6 ± 40	0.57

*ICA* index of contractile asymmetry; *, *p* < 0.05

Stratified by etiology of cardiomyopathy, relative reduction of ICA4 was greater both in responders with ischemic (− 36 ± 26% vs. 6 ± 47%, *p* = 0.001) and non-ischemic (− 43 ± 21% vs. -12 ± 18%, *p* = 0.004) cardiomyopathy.

### Correlation of baseline ICA and reduction of ICA

Baseline ICA4 was linearly correlated with relative reduction of ICA4 after CRT (*r* = 0.73, *p* < 0.0001) (Fig. 4 right upper panel). Responders with higher baseline ICA4 (≥ 0.7 s^− 1^) achieved a significantly higher relative reduction of ICA in three LV sectors (3 to 5) compared to responders with a lower baseline ICA4 (Fig. 4 left lower panel). At the same time, in the patients with a lower baseline ICA4 (< 0.7 s^− 1^), CRT response was only associated with ICA reduction in two LV sectors (4 and 5, Fig. 4 right lower panel).

### Time to peak delay and LBBB pattern

Mechanical dyssynchrony based on time to peak delay ≥ 130 ms was present in 46 (69.7%) responders and 16 (69.6%) non-responders (*p* = 0.99). Responders had classical LBBB pattern in 52 (78.8%) cases and non-responders in 11 (47.8%) cases (*p* = 0.008), providing sensitivity 79%, specificity 52%, PPV 83%, NPV 46%, and accuracy 72%.

### Logistic regression

The results of the logistic regression are summarized in Table 4. In a univariable model, CRT response was predicted by baseline ICA4, LBBB pattern, and renal function. In the multivariable models using age, renal function, and either ICA4 or LBBB pattern as predictors, ICA4 ≥ 0.7 s^− 1^ had odds ratio (OR) 10.1 (95% CI 3.2–40), and LBBB pattern had OR 4.44 (95% CI 1.54–13.6). In ROC analysis, the two models had AUC 0.8 (95% CI 0.7–0.91) and 0.72 (95% CI 0.6–0.85), respectively, *p* = 0.18 (Additional file 4).

**Table 4:** Logistic regression of predictors of response to cardiac resynchronization therapy

	Odds ratio (95% CI)	*p*-value
Univariable analysis		
Age	0.95 (0.9–1)	0.07
Male sex	0.51 (0.17–1.4)	0.21
eGFR < 60 ml/min/1.73 m^2^	0.37 (0.13–0.96)	0.04*
QRS duration	1 (0.98–1.03)	0.79
Ischemic etiology	0.59 (0.2–1.59)	0.31
Time to peak ≥ 130 ms	1.01 (0.34–2.76)	0.99
LBBB pattern	4.05 (1.49–11.4)	0.007*
ICA4 ≥ 0.7 s^− 1^	10.2 (3.35–38.5)	< 0.001*
Multivariable model 1		
Age	0.95 (0.89–1.01)	0.1
eGFR < 60 ml/min/1.73 m^2^	0.45 (0.15–1.26)	0.13
LBBB pattern	4.44 (1.54–13.6)	0.007*
Multivariable model 2		
Age	0.95 (0.89–1.01)	0.12
eGFR < 60 ml/min/1.73 m^2^	0.55 (0.18–1.69)	0.3
ICA4 ≥ 0.7 s^−1^	10.1 (3.2–40)	< 0.001*

*ICA4* Index of contractile asymmetry in left ventricular sector 4, *eGFR* estimated glomerular filtration rate, *GLS* Global longitudinal strain, *LBBB* Left bundle branch block; *, *p* < 0.05

### Intraobserver analysis

ICA from the six LV sectors showed a good intra-observer agreement with intraclass correlation coefficient 0.89 (95% CI 0.82–0.93) and bias − 0.04 s^− 1^ (95% CI -0.23-0.15). Coefficient of variation of ICA was 16.7%.

## Discussion

In this study of patients having LBBB and HF, the presence of contractile asymmetry in entire opposing LV walls measured as SD of differences in strain rate throughout systole (ICA) was associated with an increased likelihood of response to CRT. QRS duration, which is the mainstay for patient selection according to the current guidelines [10], did not have any additional value in predicting response after CRT. Both baseline ICA4 and improvement in ICA4 exhibited a linear correlation with the degree of reverse LV remodeling. Moreover, the ability of ICA to predict response to CRT was present both in case of ischemic and non-ischemic cardiomyopathy. Classical LBBB pattern, but not time to peak delay was also found to predict response to CRT.

At current stage, the three CAMM plots from the standard apical views had to be saved manually as individual image files. Otherwise, besides traditional post-acquisition STE analysis of 2D echocardiograms, no further undertaking by the user was necessary in terms of image handling. Based on the data contained in the CAMM plot images, the fully automated calculation of ICA values in the six LV sectors was feasible and could be performed in less than 1 min in each patient. The rationale behind extending the ICA analysis beyond the three standard apical views and dividing LV into 12 sector pairs is uneven distribution of contractile asymmetry as demonstrated in Fig. 5.

In the subgroup of patients with a low baseline ICA4, who did respond to CRT, ICA improved more compared to non-responders in two LV sectors ranging from 91 to 150 degrees. Meanwhile, in responders with higher ICA4, both response rate to CRT and the degree of relative reduction of ICA was higher. The association between CRT response and improvement of dyssynchrony assessed by ICA is in line with previous studies demonstrating increased likelihood of CRT response in case of dyssynchrony reduction and worse outcome in case of induced dyssynchrony after CRT [17].

Echocardiography is a part of routine patient assessment before CRT. However, this use of echocardiography is limited to the assessment of LVEF ≤ 35%. Currently, QRS morphology and width are used to assess an abnormal electrical activation for the patient selection. Meanwhile, numerous CRT trials have attempted to use echocardiography by addressing intraventricular mechanical activation delay in order to extend its use beyond LVEF. Initially, echocardiographic techniques using M-mode or pulsed Doppler, such as septal-posterior wall motion delay or interventricular mechanical delay were used to quantify dyssynchrony [18]. In the recent times, TDI and segmental strain imaging applying STE were most widely used and published techniques for dyssynchrony assessment. Using these methods, dyssynchrony was quantified by measuring the time-to-peak of the opposing walls [12, 19]. More recently, 3D STE strain analysis was suggested for LV dyssynchrony quantification as well, although being limited by low volume rate [20]. However, despite promising results in single-center studies, these techniques failed to improve the patient selection for CRT beyond the ECG criteria in a multi-center setting [21].

STE has been demonstrated to be more robust against angular error than TDI. Also, unlike time to peak by TDI, STE has been applied in more sophisticated dyssynchrony assessment methods such as cross-correlation analysis, classical LBBB pattern, or 3D activation pattern [4, 6, 16, 22, 23]. Along with septal rebound stretch, apical rocking, and septal flash [7, 24], they have shown the potential to identify responders to CRT in addition to ECG criteria. The current method could be an addition to the club of these new methods. However, this method is very elaborate and uses more than > 160 data lines from each myocardial wall in the apical views proving a thorough assessment of contractile asymmetry between the myocardial walls and a high predictive value for CRT response.

Myocardial deformation parameters, including strain rate, are load dependent [25]. Recently, assessment of myocardial work based on pressure-strain loops incorporating both STE and LV pressure, has shown promising results in predicting CRT response [26, 27]. Calculation of myocardial work involves multiplying regional strain rate by LV pressure [26]. While LV pressure remains constant determined by arterial blood pressure, the actual variability among segmental work is due to segmental differences in strain rate. ICA might therefore be regarded as a simplified method of assessing unbalance between myocardial work in the opposing LV walls.

This approach was taken a step further to examine LV outside the standard apical views. The interpolation provides a dynamic 3D model of the mechanical LV activation as well as permits assessment of strain rate values in any “virtual” apical view. LV sector 4 located between 91 and 120 degrees corresponding to 3 to 4 o’clock appears to be in accordance with the direction of LV activation in LBBB with an early contraction of LV septum and a pre-stretch followed by a delayed contraction of the lateral wall [28]. Moreover, sectors 4 and 5 seem to represent the typical location of the LV lead placement in the lateral or posterolateral wall [10]. This strongly suggests that the angle of optimal dyssynchrony assessment before CRT may be located outside the standard views. Similarly, the same might be true for the direction of the greatest reduction of contractile LV asymmetry after CRT.

While data on LV scar were not available, from a theoretical point of view, ICA is expected to be low in a scarred LV sector. In case only one of the opposing walls contracts while the other is stretched, the SD of the strain rate differences is expected to be low. This in turn suggests a reduced probability of response to CRT, should the lead be placed in the scarred sector.

Integration of the present analysis algorithm into the current software would allow a useful and clear visual aid that can be used to highlight mechanical LV dyssynchrony. ICA has a potential to improve the patient selection for CRT by differentiating a mechanically dyssynchronous LV responsive to CRT from a poorly contracting LV without dyssynchrony. The prognostic value of targeted LV lead placement guided by ICA would be of interest in future research. The same is true for application of ICA on images of even higher temporal resolution to gain further insights into contractile asymmetry both in healthy subjects and in disease.

### Study limitations

Although the processing of the data was performed in a freely available statistics software, the algorithm behind the generation of the original CAMM plots in the echocardiography package is currently unavailable in the public domain. Myocardial scar burden was not taken into account in this study. In addition, a more detailed clinical status of the patients, including natriuretic peptides, was not available.

## Conclusions

ICA allows a visual 3D representation of dyssynchronous contraction outside of traditional apical views. The study suggests that high pre-implantation contractile asymmetry is associated with a high responder rate. The present method can both be useful in future selection of patients for CRT implantation and may reduce the number of non-responders.

## Supplementary information

**Additional file 1.** Algorithm of conversion of CAMM plot to a matrix containing strain rate values and the principle of building a 3D strain rate model.

**Additional file 2.** Comparison of exported EchoPAC (r) systolic strain rate values and CAMM plot-based values in a three-chamber view representing the six traditional strain rate curves.

**Additional file 4.** Receiver operating characteristics plots of multivariable models containing age, renal function, and either ICA4 or classical LBBB pattern.

## Data Availability

The datasets used in the current study are available from the corresponding author on reasonable request.

## Abbreviations

AUC: Area under curve
CAMM: Curved anatomical M-mode
CI: Confidence interval
CRT: Cardiac resynchronization therapy
EF: Ejection fraction
ESV: End-systolic volume
HF: Heart failure
ICA: Index of contractile asymmetry
ICA4: Index of contractile asymmetry in left ventricular sector 4
LBBB: Left bundle branch block
LV: Left ventricle
NPV: Negative predictive value
PPV: Positive predictive value
ROC: Receiver operating characteristics
RV: Right ventricle
SD: Standard deviation
STE: Speckle tracking echocardiography
TDI: Tissue Doppler imaging
