# Supplementary material for: Index of contractile asymmetry improves patient selection for CRT: a proof-of-concept study
Source: Cardiovasc Ultrasound. 2019 Oct 10;17:19. doi: 10.1186/s12947-019-0170-2 (PMC6788085; doi:10.1186/s12947-019-0170-2)
Supplement: Supplementary file 2 — Additional file 1. Algorithm of conversion of CAMM plot to a matrix containing strain rate values and the principle of building a 3D strain rate model. [file 12947_2019_170_MOESM1_ESM.docx]

### Appendix A: Automated algorithm of conversion of curved anatomical M-mode plot to a matrix containing strain rate values and the principle of building a 3D strain rate model using cubic spline interpolation

The scale of strain rate was adjusted in every image down to the narrowest range still containing all systolic strain rate values. Curved anatomical M-mode (CAMM) plots of systolic strain rate were first saved as digital images in lossless JPEG format. Then, every JPEG image of the systolic strain rate was imported as an array containing red-green-blue (RGB) color data using the package 'JPEG' version 0.1-8 in the statistical program R version 3.3.0 (The R Foundation for Statistical Computing, Vienna, Austria). Afterwards, the RGB values of each pixel were converted to hue-saturation-value (HSV) data using the R package 'grDevices' version 3.3.0. The hue part in HSV values denotes the color value on a 360-degree scale, e.g. red is represented by 0 and 360, yellow is 60, green is 120, blue is 240.

Based on the color palette provided in EchoPAC software, the green color in the CAMM plots of strain rate represents 0 s^-1^. The positive part of the strain rate scale extends from cyan (180 degrees in the HSV scheme) to blue (240 degrees in the HSV scheme). The negative scale lies between yellow (hue value 60) to red (hue value 0). Knowing the range of the strain rate scale in the individual speckle tracking echocardiography analysis (i.e. which maximal strain rate value is coded blue and which minimal strain rate value is coded red), the pixel wise decoding of the image was performed. For instance, in case of a strain rate scale of $\pm$ 1.8 s^-1^, a hue value 30 corresponds to -0.9 s^-1^, hue 0 to -1.8 s^-1^, hue 220 to 1.2 s^-1^, hue 240 to 1.8 s^-1^ etc.

**The principle behind the generation of strain rate values outside of the three standard apical views using cubic spline interpolation**


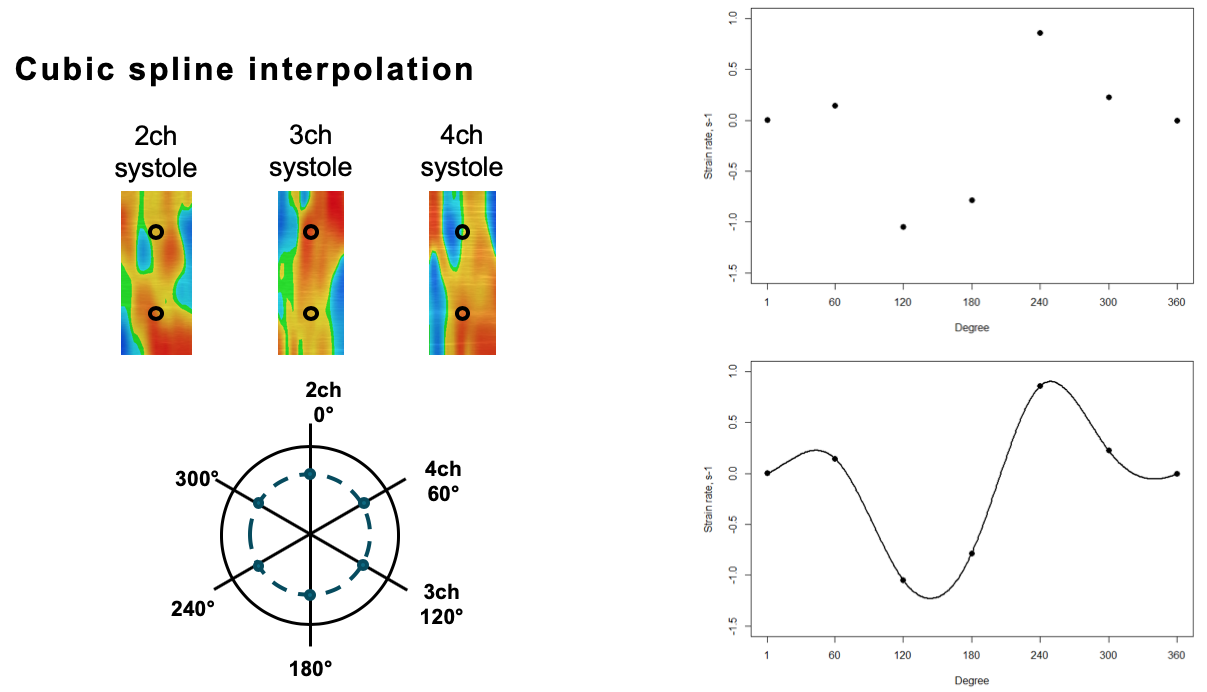


The circles on the CAMM plots (upper left panel) represent the geometrical location of the known systolic strain rate values in the standard apical views and are also marked by the dots in the lower left panel. The upper right panel shows the degrees around left ventricle in the clock-wise direction on the x axis, and absolute strain rate values on the y axis. Note that there are seven and not six dots in the plot. This is due to the fact, that the first and the last dots represent the same point in the anterior wall (0 degrees and 360 degrees). The interpolated curve in the lower right panel corresponds to the strain rate values geometrically represented by the dashed line in the lower left panel.

As this figure is presented for the illustration purposes only, the absolute strain rate values in the plots on the right-hand side panels do not correspond to the strain rate values in the particular CAMM plots shown in the upper left panel.
