# Supplementary material for: Index of contractile asymmetry improves patient selection for CRT: a proof-of-concept study
Source: Cardiovasc Ultrasound. 2019 Oct 10;17:19. doi: 10.1186/s12947-019-0170-2 (PMC6788085; doi:10.1186/s12947-019-0170-2)
Supplement: Supplementary file 3 — Additional file 2. Comparison of exported EchoPAC (r) systolic strain rate values and CAMM plot-based values in a three-chamber view representing the six traditional strain rate curves. [file 12947_2019_170_MOESM2_ESM.docx]

### Appendix B: Comparison of exported EchoPAC (r) systolic strain rate values and CAMM plot-based values in a three-chamber view representing the six traditional strain rate curves.

The EchoPAC (r) screenshot of the source image is provided below. CAMM, curved anatomical M-mode.
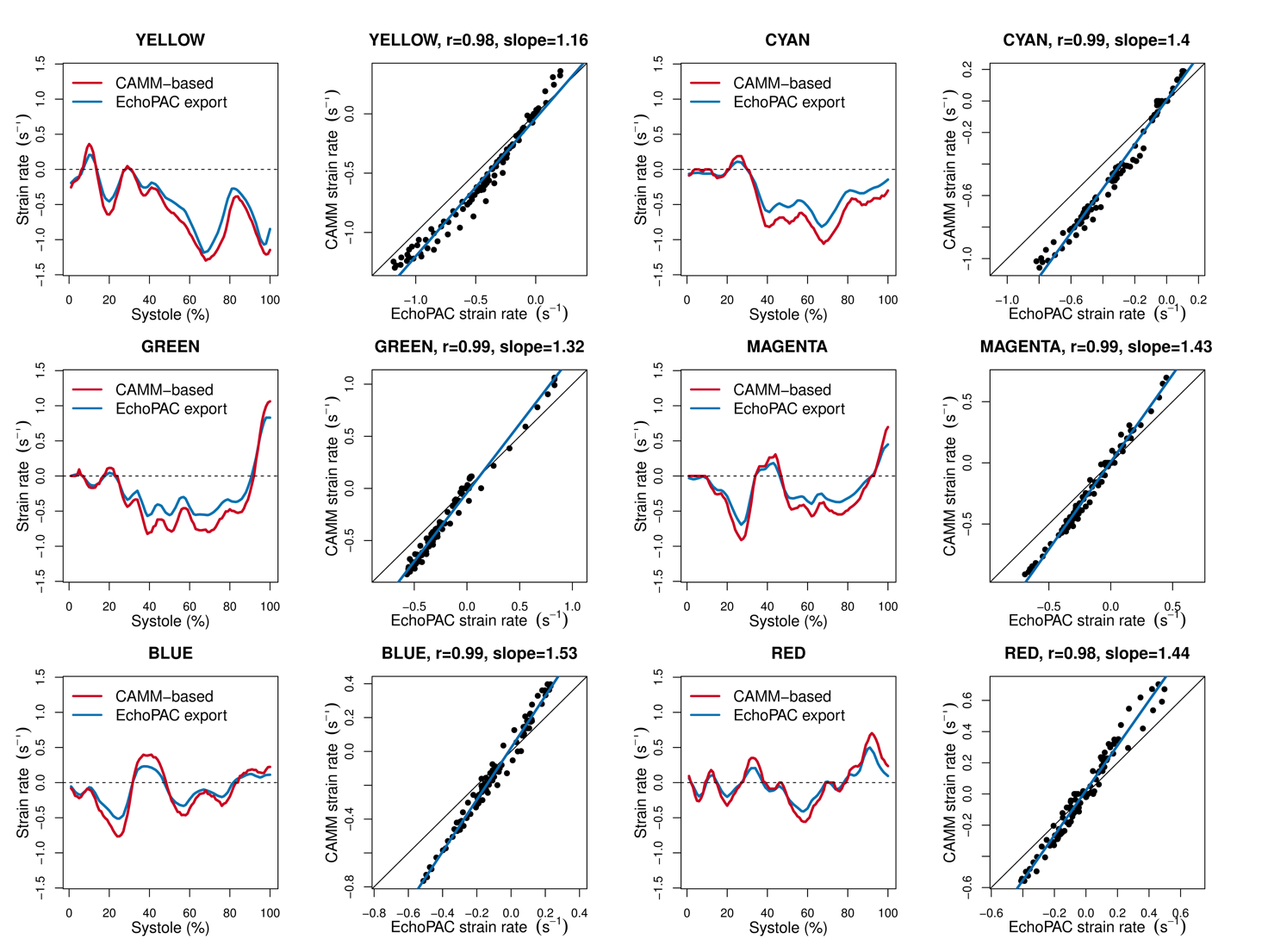


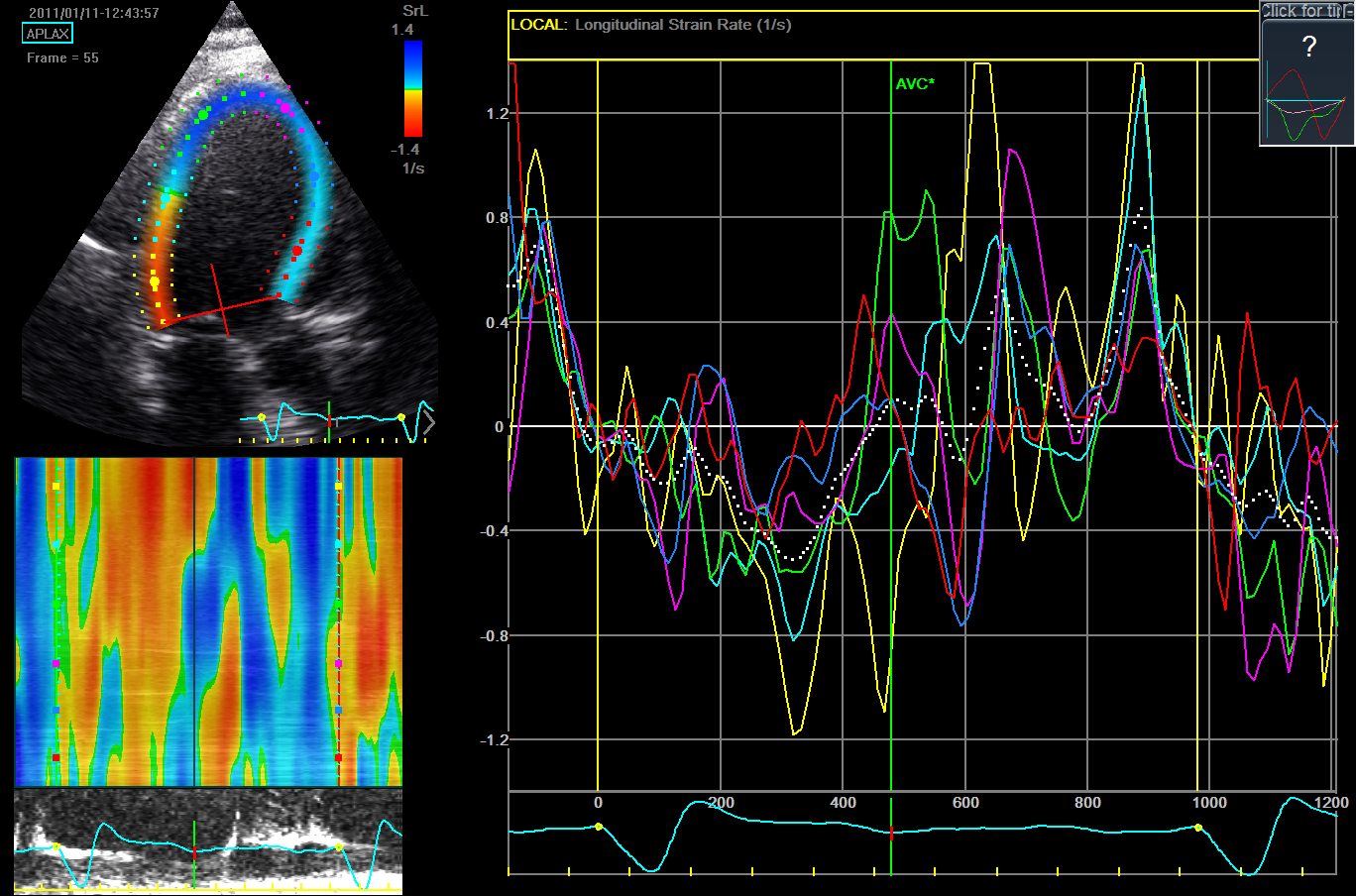


Index of contractile asymmetry in the three-chamber projection in this case was 0.51 s^-1^.
