# Supplementary material for: Index of contractile asymmetry improves patient selection for CRT: a proof-of-concept study
Source: Cardiovasc Ultrasound. 2019 Oct 10;17:19. doi: 10.1186/s12947-019-0170-2 (PMC6788085; doi:10.1186/s12947-019-0170-2)
Supplement: Supplementary file 4 — Additional file 4. Receiver operating characteristics plots of multivariable models containing age, renal function, and either ICA4 or classical LBBB pattern. [file 12947_2019_170_MOESM4_ESM.pdf]

Appendix E: Receiver operating characteristics plots of multivariable models containing age, renal function, and either ICA4 or classical LBBB pattern.

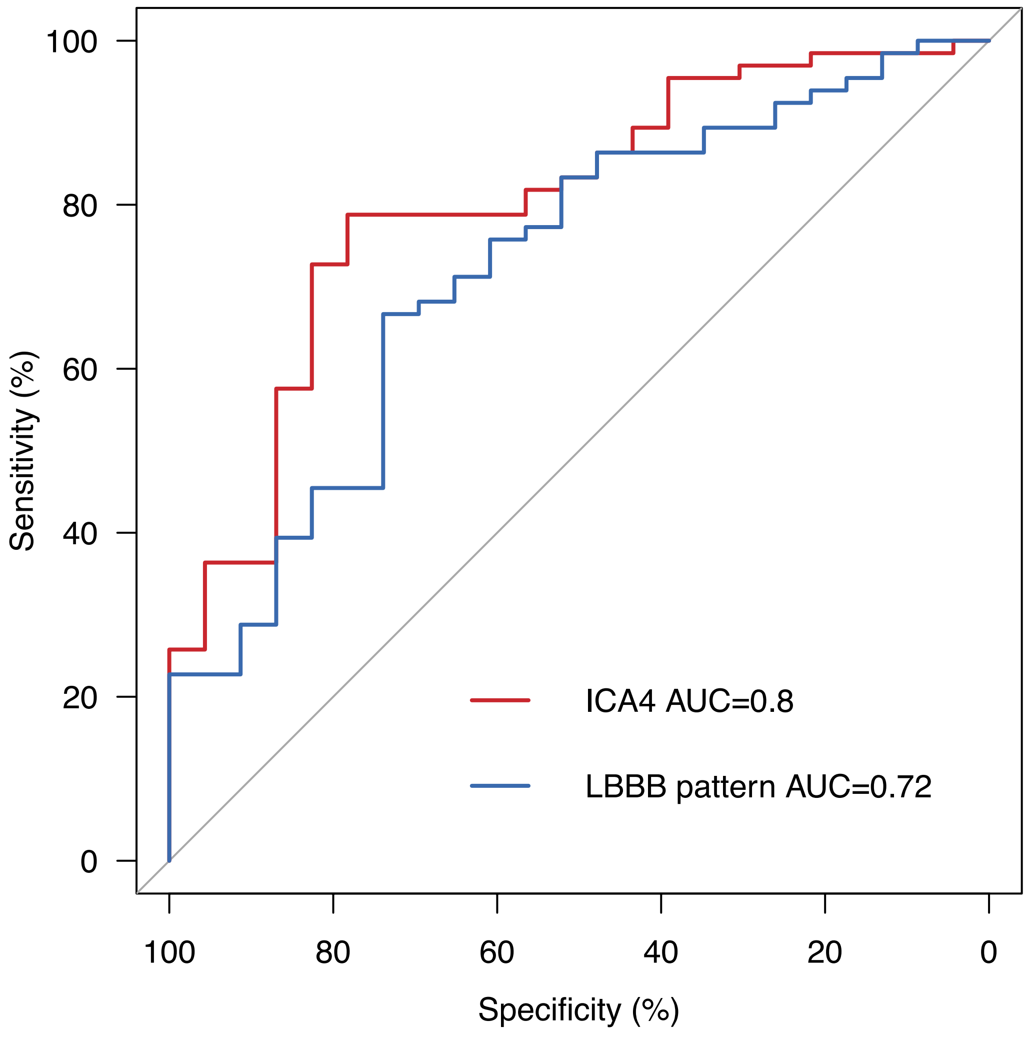

AUC, area under curve; ICA4, index of contractile asymmetry in left ventricular sector 4; LBBB, left bundle branch block.
